# Child abuse and neglect in the Jaffna district of Sri Lanka – a study on knowledge attitude practices and behavior of health care professionals

**DOI:** 10.1186/s12887-018-1138-3

**Published:** 2018-05-05

**Authors:** M. G. Sathiadas, Arunath Viswalingam, Karunya Vijayaratnam

**Affiliations:** Department of Paediatrics, University of Jaffna, PO Box: 57, Adiyapatham Raod, Jaffna, Sri Lanka

## Abstract

**Background:**

Victims and perpetrators of child abuse do not typically self-report to child protection services, therefore responsibility of detection and reporting falls on the others. Knowledge on child protection is essential for the first contact person and such information is sparse in research literature originally coming from Sri Lanka. Anecdotally, several cases of child abuse have been missed out at the first contact level. Therefore we undertook this survey to assess the knowledge, attitudes towards child protection and the experiences of medical officers, nursing officers and social workers on child protection.

**Method:**

This was a descriptive analytical study carried out in hospitals and the community during March–October 2016. An anonymous content validated self-administered questionnaire was used as the study instrument. Knowledge, Attitude, Practices and Behaviour were assessed via multiple choice questions and responses according to Likert score. Three anonymised case records were given as case vignettes to be studied by the participants and their responses were also recorded on the questionnaire.

**Results:**

Among the 246 responders 156 (63.4%) were doctors. All groups of professionals identified the forms of child abuse correctly and the social indicators of child abuse was correctly identified in 152 (61.7%). Majority failed to identify the features of the perpetrator. Majority of the professionals showed a favourable response in attitude when dealing with child maltreatment. 153 (62%) had suspected child abuse in their career and 64% of them had reported it to the authority. Fifty two (21%) had attended a training workshop on child abuse and 65.8% of the responders were not satisfied with their knowledge. 229(93%) of them indicated that they wanted some form of education on child maltreatment. The Knowledge, Attitude and Behaviour towards child abuse were significantly good on people with experience in the field of Paediatrics and Judicial Medical work, when compared to those who did not have the experience in these two fields. (*p* value< 0.01).

**Conclusion:**

Although the knowledge among health professionals regarding child abuse and care was satisfactory, further areas need reinforcement. The attitude was more positive, the behavior and practices on child maltreatment needed reinforcement via workshops and continuing medical education.

## Background

Child Maltreatment or Abuse has been a worldwide problem and continues to be a major crisis in our current society as well. Child maltreatment is defined by the World Health Organisation (WHO) as abuse and neglect that occurs to children under 18 years of age. It includes all types of physical, sexual abuse, neglect and negligence of the child, emotional ill treatment and exploitation for commercial and non-commercial reasons. This can lead to problems in child’s health, survival and dignity of the child especially in responsibility, trust and power [1]. The National Society for the Prevention of Cruelty to Children also describes the types of abuse similarly to the WHO [2].

The physical signs of abuse may include unexplained bruising, marks or injuries on any part of the body, multiple bruises which are unexplained, cigarette burn marks, broken bones and scalds, with upward splash marks [2]. Changes in behaviour can also indicate physical abuse. The symptoms can be child’s fear anticipating the parents being approached for an explanation by the autorities, aggressive behaviour or severe temper tantrums, flinching when touched, depression and withdrawn behaviour [2].

Visible evidences are seen in physical abuse whereas these are absent in emotional abuse or neglect but can leave deep, long lasting scars in their minds. When abused children get help early, their chances of recovery and healing from it is greater [3].

Most common forms of child abuse have been recorded in South Asian region and in addition to them, conscription of children during armed conflict, which is a new form of child abuse, has also been recorded especially in Sri Lanka and Nepal [4]. The 2006 UN Study on Violence against Children, estimated that in South Asia, between 41 and 88 million children witness violence at home every year. Evidence also indicates that half of the world’s child brides live in South Asia, where 46% of women aged 20–24 are first married or in union before they reach the age of 18 and that around 44 million children are engaged in child labour across the region. Sexual abuse and exploitation, as well as child trafficking and corporal punishment raise additional concerns in the region. No data are provided on sexual abuse and exploitation, despite the importance of these phenomena in the region. Abuse is often not reported and is shrouded in secrecy; hence the regional data is scarce [5].

The reports on child abuse, neglect and exploitation are increasing in Sri Lanka as well. According to the figures tabled in the Sri Lankan Parliament in April 2013, there are about 15,000 legal trials pending Nationwide and more than 4,000 (27%) involve some form of violence towards a child. Around 1500 cases per year are reported for issues related to children. The same report says there were 1,750 cases of child rape, 5,475 cases of child molestation and 1,194 cases of child abuse in 2012 [6]. The number reported is much less than the actual incidence, because large number of children do not report abuse [6].

Another major reason for underreporting of child maltreatment in this region is due to the sociocultural impact it makes as most of the abuse victims are alienated and hence do not get reported. Primary caretakers from Asian countries were less likely to report the abuse to authorities when compared to the other caretakers [7, 8]. Most of the primary caretakers from Asia disbelieved child abuse, hence the children do not self-report to the carer. Discussing family problems to anyone outside the family can be considered shameful. The cultural pressures makes the victim to internalise the conflict and they are least supported. They express more suicidal impulses rather than anger and hostility [8].

Diagnosis and management of child abuse is a challenge and has to be done through a multidisciplinary approach involving medical and legal professionals. Therefore, precise knowledge on the subject of child maltreatment is essential among these professionals [9].

Events of child abuse and neglect are commonly not detected as first responders in child care fail to identify injuries, conditions, or behaviours. In the absence of timely intervention, an abused child has a 10% risk of having fatal injuries [10]. A study done in Central Gujarat India suggests that medical and dental residents are not prepared in detecting and managing children with issues related to protection [11]. A significant gap was seen in recognising and responding effectively. Mandated training on detecting and management of child abuse and neglect, to all medical and allied professionals will improve reporting of suspected child abuse [12].

It is known that victims and perpetrators of child abuse do not usually self-report to child protection services [5] Medical officers, being the first responders in most cases, are in an ideal position to report abuse. Hence, it is very important for medical officers to be familiar on medico-legal aspects of child abuse.

Therefore, the objective of this study was to describe the knowledge, attitudes and experiences of medical officers, nursing officers and social workers regarding child abuse in the Jaffna District of Sri Lanka, and to assess the associations with socio demographic factors, experience in the field of Paediatrics and Judicial Medicine towards child abuse.

## Methods

### Study design

This was a descriptive analytical study which was carried out in hospitals and community in the Jaffna District of Sri Lanka from March to October 2016.

### Setting

The Jaffna District, Sri Lanka is situated in the North of Sri Lanka and has one tertiary care centre and three general hospitals. The tertiary care centre has specialists care and receives referrals from all the general hospitals. All these hospitals together, cater for the entire population of 610,640. [13] and would see approximately 200,000 per annum at the outpatient and emergency departments. Children seen for child abuse at the peripheral units also get referred to the tertiary unit for specialised care.

### Participants

Simple random sampling was done among the doctors, nurses in all 4 hospitals and social workers from the community. This included, all the medical and dental officers (includes Consultants, Senior Registrars, Registrars, Senior House Officers (SHO), Resident House Officer (RHO), Intern Medical Officers (IMO)) and Nursing Officers working in different hospitals. Social workers are personals who work mainly in the community and play a role in identifying child abuse and neglect in the field.

Sample size was calculated using the Daniel [14] formula and the p was 13% according to Starling et al. [15]. Level of confidence was 95% with z being 1.96 and the non-response rate was 20%. This gave a total sample size of 208, enough to obtain a 95% confidence interval that the results could be generalised to a wider population [15].

As there was definite sampling frame, a simple random sampling was done. Eligible sample of participants were informed and a written informed consent was obtained. Ethical approval was obtained from Faculty of Medicine, University of Jaffna, Sri Lanka. (J/ERC/16/72/NDR/0143).

### Data collection and analysis

An anonymous pre-tested and standardised self-administered questionnaire was used as the study instrument. The questionnaire included questions to assess the socio-demographic factors of medical, nursing and social workers. The 24-item questions were in the native language comprising of multiple choice or true false format based on the literature, to assess the knowledge, and a 10 item questions to assess the attitude. A field test was conducted with 10 experts in the field of child abuse to measure the content validity. Content Validity Ratio (CVR) was calculated using Lawshe’s formula CVR = (N_e_ - N/2)/(N/2), in which the N_e_ is the number of panellists indicating “essential” and N is the total number of panellists [16]. The CVR for the whole questionnaire was 0.80. The questionnaire was modified as per the expert suggestions, and the modified version was used as the study tool.

Three case vignettes were prepared from anonymised case records. The cases were physical abuse, sexual abuse and neglect based on delay in seeking medical help. These case histories were given in paper format and confidence in the story was assessed on a 5 point scale (1 being not confidant and 5 being very confident) and reporting and taking action on the individual cases was assessed by “yes”/ “no” responses. Cronbach alpha was used to assess the reliability of the scores.

To assess the knowledge, questions regarding types of child abuse, identifying features of abuse and characteristics of the perpetrator were considered. Questions were analysed by responses to each of the question separately and were expressed as percentage. Chi-square test for significance of difference among proportions was calculated.

To assess the attitude of the participants regarding child abuse and neglect, each respondent was asked 10-item questions.. Responses were recorded as “strongly agree” or “disagree”, or “somewhat agree” or “disagree”, or “don’t know”/ “can’t say”. Depending on whether it was a proper attitude or not, scores from 1 to 4 were allotted. A score of ‘0’ was given for “don’t know”/ “can’t say”. Six items had negative statements and they were allocated the reverse scores. A total 40 was then divided into sub scores which were defined as 0–9 Very Poor, 10–18 considered as there are many issues which need changing, 19–27 as more positive attitudes and 28–40 as having a good overall attitude. Analysis of variance for significance of difference among means was calculated and Cronbach alpha was used to assess the reliability of the scores.

To assess the experiences of participants, five questions in the questionnaire were provided and the responses were expressed as percentage. Each respondent was asked if s/he undertook any particular action in the previous year that would help towards having better practices.

The data was described using frequencies and percentages. *P* value of < 0.05 was considered as statistically significant. Data was coded and entered in SPSS version 20.

## Results

A Total of 273 were selected for the survey and number of responders was 246(90.1%). Among the responders, 156 (63.4%) were medical officers, 59 (24%) were nursing officers and others were social workers. Mean age of the subjects was 34.70 ± 7.924 yrs. Male female ratio was 1:1.29. Most of them (149–60.6%) were married, 108 (43.7%) had experience less than 5 years in their respective fields and 107 (43.5%) had children of their own. The characteristics of the responders along with their experiences in the profession are provided in Table 1. Among those in the medical profession 33(21.2%) were intern medical officers, who were the first contact in most of the cases when a patient is admitted, 19(12.2%) were consultants and 104(66.7%) belonged to the middle grade, whose experience varied from 1.5–15 years.

**Table 1:** Characteristics of the responders and their experience

Feature		Medical officers (*N* = 156)	Nursing officers (*N* = 59)	Social and probation (*N* = 31)
Age (years)	26–30	73(46.8%)	26(44.1%)	2(6.4%)
	31–35	36(23.1%)	11(18.6%)	11(35.5%)
	36–40	21(13.5%)	4(6.8%)	10(32.3%)
	> 40	26(16.7%)	18(30.5%)	8(25.8%)
Gender	Male	77 (49.4%)	18 (30.5%)	12 (38.7%)
	Female	79(50.6%)	41(69.5%)	19(61.3%)
Experience (years)	0–4.9	76(48.7%)	22(37.3%)	10(32.3%)
	5–9.9	36(23.1%)	16(27.1%)	10(32.3%)
	> 10	44(28.2%)	21(35.6%)	11(35.5%)
Marital Status	Single	67(42.9%)	23(39%)	7(22.6%)
	Married	89(57.1%)	36(61%)	24(77.4%)
Having children	Yes	62(39.7%)	26(44.1%)	19(61.3%)
	No	94(60.3%)	33(55.9%)	12(438.7%)
Experience in paediatrics and judicial work	Yes	69(44.2%)	21(35.6%)	0(0%)
	No	87(55.8%)	38(64.4%)	0(0%)

### Knowledge of the responders

All groups of professionals were able to identify the forms of child abuse correctly and there was no significant difference between the groups, except on seeking timely medical advice. Frequency of identifying the types of child abuse is provided in Table 2.

**Table 2:** Frequency of identifying type of abuse

Type of child abuse or neglect	Medical officers (N = 156)	Nursing officers (N = 59)	Social workers (N = 31)	X^2^ (*p* value)
Failure to seek needed medical treatment	148(94.9%)	39(69.6%)	30(96.8%	36.58 (p = 0.000)
Neglect of child education	147(94.2%)	53(89.8%)	31(100%)	3.751 (*p* = 0.153)
Beating causing injury	139(89.1%)	55(93.2%)	31(100%)	4.240 (*p* = 0.120)
Non-injurious spanking	110(70.5%)	39(66.1%)	16(51.6%)	4.216 (*p* = 0.121)
Verbal humiliation	139(89.1%)	54(91.5%)	31(100%)	3.792 (*p* = 0.150)
Sexual abuse	148(94.8%)	57(96.6%)	31(100%)	1.835 (*p* = 0.400)

The knowledge of the social indicators of child abuse was correctly identified by 152 (61.7%). The knowledge on the features of the perpetrators was satisfactory in 74%(*n* = 182). The knowledge of the perpetrator being known to the family was identified in 75%(*n* = 187), perpetrator being abused as a child in 62%(*n* = 153) and having a psychiatry background in 61%(*n* = 152). There was no significant difference between the groups in the identifying features of the perpetrators. (*p* value > 0.5).

Knowledge of the physical indicators was satisfactory in all groups of health workers. (Table 3).

**Table 3:** Knowledge of the physical indicators

Question	True	False	Not responded
Bruising over bony prominence is an indication of abuse	142(57.7%)	98^a^(39.8%)	06(2.4%)
Burns are associated with abuse	148^a^(60.1%)	90(36.5%)	08(3.25%)
Bite marks on the shoulder be investigated for child abuse	107^a^(43.49%)	79(32.1%)	60(24.3%)
Child expresses fear of going home after a period in the hospital	213^a^(86.5%)	31(12.6%)	02(0.8%)
A history that is vague and defers each time tells it is a possible indicator of abuse	172^a^(69.9%)	72(29.2%)	02(0.8%)
Torn frenulum indicates child sexual abuse	76(30.9%)	68^a^(27.6%)	102(41.4%)
Sexualised behaviour in the child may be due to sexual abuse	146^a^(59.5%)	97(39.4%)	03(1.2%)

^a^correct answer

The three case vignettes were mainly of child physical abuse, sexual abuse and seeking delayed medical help (Neglect). All categories of people identified the type of abuse correctly. Cronbach alpha was 0.81 in confidence in reporting, suggesting good internal consistency. The mean scores for confidence in reporting were 2.7, 3.1 and 1.4 for cases of physical, sexual abuse and neglect respectively. The differences in the mean scores also had a significant difference between the groups. (*P* value < 0.001) The decision to report to the authority was 90% (*n* = 222) in the case of physical abuse, 97% (*n* = 240) in sexual abuse and 65% (*n* = 160) in the case of neglect. The decision to report was significant between the groups in dealing with the case of neglect (*p value < 0.001*) but not in physical and sexual abuse. (*p values > 0.5*) Experience in the field of paediatrics and judicial work did not have a significant association in identification and reporting of the cases given in the case vignettes.

The source of knowledge was mainly through the university education system (54.1%) followed by reading the literature (52.8%) and following Continuing Medical Education (CME) programme on child abuse (41.1%).

### Attitude of the responders

Mean attitude scores were 20.16 ± 3.3, 20.25 ± 4.04, 23.84 ± 5.3 for doctors, nurses and social-workers respectively. (F = 12.55 *p* = 0.000) Even though the majority of the professionals showed a more positive attitude, there are many issues that need changing. (Table 4) Majority (76.4%) were confident in reporting child abuse and 24% said they would defer reporting until firm evidence was present. 60.5% were confident in giving evidence in a court of law and 45% were not familiar with the legal issues. Only 24.3% were satisfied with the local child protection services (Table 5).

**Table 4:** Attitude scores in health professionals

	Medical officers (*N* = 156)	Nursing officers (*N* = 59)	Social workers (*N* = 31)	Total	Statistical analysis
Mean attitude score	20.16 ± 3.3	20.2 ± 4.0	23.84 ± 5		F (2,243) = 12.55 *p* = 0.000^a^
Issues need changing (10–18)	51(32.7%)	17(28.8%)	8(25.8%)	76(30.9%)	X^2^(4,*N* = 246) = 30.3 *p* = 0.001^b^
More positive attitudes (19–27)	101(64.7%)	41(69.5%)	15(48.4%)	157(63%)	
Good feeling overall (28–36)	4(2.6%)	1(1.7%)	8(25.8%)	13(5.3%)	

^a^Analysis of variance for significance of difference among means

^b^Chi-square test for significance of difference among proportions

Crohnbach alpha 0.74

**Table 5:** Attitude towards child abuse

Attitudes	Positive Responses			ANOVA
	Medical officers (N = 156)	Nursing officers (*N* = 59)	Social workers (*N* = 31)	
Professional is responsible for the stigma that occurs to the family^b^	74(30.1%)	27(11.0%)	11(4.5%)	F(2,243) = 4.933 (*p* = 0.008)
Confidence in reporting child abuse^a^	112(45.5%)	50(20.3%)	26(10.6%)	F(2,243) = 1.443 (*p* = 0.238)
Confident in giving evidence in a court of law^a^	93(37.8%)	33(13.4%)	23(9.3%)	F(2,243) = 1.443 (*p* = 0.136)
Not familiar with the legal issues^b^	77(31.3%)	27(11.0%)	7(2.8%)	F(2,243) = 6.220 (*p* = 0.002)
Reported only if persistence of abuse^b^	19(7.7%)	6(2.4%)	3(1.2%)	F(2,243) = 1.548 (*p* = 0.215)
Defer reporting if no firm evidence^b^	43(17.5%)	10(4.1%)	6(2.4%)	F(2,243) = 7.344 (*p* = 0.001)
Satisfaction with the local child protection services^a^	27(11.0%)	20(8.1%)	13(5.3%)	F(2,243) = 3.580 (*p* = 0.029)
The child should be removed from home and familiar surroundings^b^	19(7.7%)	30(12.2%)	8(3.3%)	F(2,243) = 16.422 (*p* = 0.000)
Change the school is advised in abused children^b^	44(17.9%)	33(13.4%)	11(4.5%)	F(2,243) = 6.803 (*p* = 0.001)

^a^Positive attitude

^b^Negative attitude

### Practices and behaviour

Majority of the professionals (62%) suspected child abuse in children and only 64% had reported child abuse to the authorities previously. All the cases suspected were not reported to the authorities and the main reasons provided being: Lack of adequate history and evidence (56,6.1%), uncertainty of the diagnosis (55, 22.3%), possible harmful effects on the child’s family (31, 12.6%), lack of knowledge of the referral procedure (25, 10.1%), aggressive and angry parents (15, 6.1%), possible effect on my professional career (13, 5.28%) and fear and anxiety of the court proceedings (11, 4.47%).

All the professionals indicated that education on child protection is essential but only 52(21%) had attended training workshops on child abuse. Different practices adopted by the professionals are provided in Table 6.

**Table 6:** Practices and behaviour of the professionals in child protection

	Medical officers (*N* = 156)	Nursing officers (*N* = 59)	Social workers (*N* = 31)	X^2^ (*p* value)
Suspect CAN	96(61.5%)	28(47.4%)	28(90.3%)	15.827 (*p* = 0.001)
Report CAN	74(47.4%)	23(39.0%)	27(87%)	20.324 (*p* = 0.001)
Aware of process of reporting	114(73.1%)	38(64.4%)	28(90.3%)	6.955 (*p* = 0.031)
Awareness of Sri Lankan laws	92(60.0%)	22(37.3%)	23(74.2%)	13.080 (*p* = 0.001)
Importance of child abuse education	156(100%)	58(98%)	31(100%)	4.743 (*p* = 0.315)
Attended training on CAN	13(8.3%)	16(27.1%)	23(74.2%)	68.956 (*p* = 0.001)
Self-satisfaction of knowledge	45(28.8%)	21(35.6%)	18(58.1%)	9.890 (*p* = 0.007)
Wish to improve the knowledge	150(96.2%)	54(91.5%)	25(80.6%)	9.964 (*p* = 0.010)

Analysis of the data showed knowledge regarding child abuse (p 0.001), knowledge of the characteristics of the perpetrator (p 0.04), attitude of more positivity towards Child abuse and neglect (*p* value 0.01), behaviour of detecting and reporting of child abuse and neglect (p 0.001) and the awareness of the law of child protection had a significant differences with the experience of the person and the speciality of paediatrics and judicial medical work. The inexperienced felt that the doctor was responsible for the stigma that occurred to these children (p 0.001).

The participants have indicated that the preferred methods of updating the knowledge on child abuse and neglect were to undertake continuing education and workshops on child abuse (70.3%) followed by information booklets (48%) and online self-study (28.5%).

## Discussion

Our study aimed at identifying the knowledge and practices of professionals, first in contact with the children who have been abused and neglected. Our study indicated satisfactory overall knowledge and it correlated well with the experience and speciality of the responders. Awareness and Basic knowledge on child abuse and neglect are the important prerequisite for reporting suspected cases of child abuse. The ability to detect and diagnose when an abused child presents, is also vital to the care of the child. When compared to studies done in Gujarat and Karnataka in India, our study indicates overall knowledge is satisfactory [11, 17].

The knowledge regarding indicators of abuse was unsatisfactory as only 68(27.6%) answered all seven responses correctly. The torn oral fraenum was identified as a form of abuse earlier but in current literature it has been disproved [18]. In this study the torn fraenum was identified as a form of abuse by 76% of the responders. This study indicates that there may be a deficiency of updated knowledge about changes to these concepts. Thomas et al. [19] have explained that this lack of updated knowledge may arise from the failure of reinforcement in the clinical setting of knowledge gained in the undergraduate classroom.

The knowledge among the medical doctors and nurses was higher when compared to the social workers about various types of abuse and physical indicators. Even though it was statistically not significant, the community social workers should have adequate knowledge of child abuse for early detection, which would prevent detrimental effects [20].

The perpetrators of Child Sexual Abuse (CSA) are usually known to the family and may have been also abused as child. This fact should be understood clearly by all healthcare professionals to prevent future perpetrators from initiating abuse [21]. In our study 23% (57) did not know the characteristic features of the perpetrators. Hence further training is needed in this aspect.

The responses to the case vignettes highlight the current knowledge and course of action. Case vignettes on physical and sexual abuse the responders were confident and the course of action of reporting was 90.2% in physical abuse and 97.5% in sexual abuse. Van Haeringen et al. [9] stated that only 69% of the health professionals reported the highest level of suspected physical abuse where as our study indicated the opposite. Case vignette of a delay in seeking medical help and neglect had the minimum score with minimum number (65%) reporting it to the authorities.

The keenness to improve the knowledge has been shown by all the professionals by indicating their interest to improve their knowledge base and interest in attending a continuing medical programme and workshops on Child abuse. The interest to be trained is a good initiative to detect child abuse and possibilities towards future introduction of screening for child abuse and neglect at emergency and outpatient settings [22].

The attitude towards more positive and good were seen in 69% of the responders and this is similar (65.5%) to a study done in Karnataka by Kirankumar et al. [17]. Attitudes towards reporting child abuse are another aspect that was studied. A suspected case of child abuse and neglect has to be reported to the authorities without delay but this can be traumatic to the parents, carers and the health care professionals. Since the stigma involved in abuse is profound in this part of the world where reporting can be a serious issue. Our study also indicated that the responders had suspected but not reported due to various reasons. This may have been due to lack of adequate history and evidence, uncertainty of the diagnosis, possible harmful effects on the child’s family, lack of knowledge of the referral procedure, aggressive and angry behaviour of parents, possible effect on the profession and fear and anxiety toward possible court proceedings. Similar fears were also noted in the study done by Deshapande et al. in Gujarat [11].

A study done by Jones et al. [23] on child abuse reporting experience, found that even at the highest levels of suspicion, only 73% of injuries were reported to child protective services and many factors hindered the reporting. This includes the clinician’s level of closeness with the family, some issues in the history and the expectations of the child protection services. In our study the health professionals indicated they were either not satisfied with the services or were not aware of the availability of the services. Training and continuing medical education along with a support system to overcome the fears can alleviate this problem. In addition, strengthening the child protection services and making health providers aware of the existence of the services, can improve child abuse being detected and reported early.

Behaviour and practices by the health care professionals towards child abuse also plays a major role in identifying cases of abuse. The need to improve the knowledge is clearly stated in our study which indicates the professionals are committed to learn and improve the services rendered. Our study found several gaps especially in the reporting system and the awareness of the existing law. All felt the importance of the issue as 99.5% indicated that education regarding child abuse was important. Even though they felt the importance, only 21.1% had attended a workshop or CME on child abuse and neglect. This was also identified in the study by Reiniger et al [12]. Modern methods of CME like on-line courses may be more appropriate in this fast moving modern world.

Self-awareness of the level of knowledge on child abuse and neglect is important for further improvements in knowledge. Our study states that 65.8% of the responders were not satisfied with their knowledge and 93% of them indicated they wanted some form of CME on abuse. It is reported that physicians in rural regions of Austria possessed basic knowledge on child abuse but were not aware of the referral system [24]. To improve diagnosis, reporting, strengthening the interaction with experts and to reduce fears in handling child abuse victim, better training is needed.

In regards to the practices of child abuse, some responders had misbeliefs, mainly in the aspect of removing the child from home (63%) after the incident irrespective of the situation that it took place. They also indicated to remove the child from the school as the family may face social isolation (35.7%). This practice can be detrimental to the child. Significant and lifelong adverse effects on the child’s mental health and development are seen in all forms of abuse. Support is needed not only medically, but also in psychosocial aspect, for the speedy recovery of the child. A child’s experience of maltreatment may cause great stress and disruption in the family and making them feel guilty about what has already occurred in the home. There is a chance that other members in the family too may have been affected. Health professionals also feel the stigma in the family and society and thereby inappropriate decisions like moving the child away from home and school has been suggested as a way of management.

Experience in the field of paediatrics and Judicial medical work indicated that the knowledge, attitude and behaviour towards child abuse and neglect were good when compared to the professionals in other specialities. As the experienced person is not the first contact person, it is mandatory to train the first responder on child abuse [8].

This study has few strengths and limitations. The random sampling technique has minimised the selection bias. The questionnaire was self-administered hence the issues that arise from face to face were overcome. The questionnaire was tested for content validity, but we were not able to perform the Pearson’s product moment correlation coefficient as the field experts were contacted only once, and the time tested second administration was not performed due to practical difficulties and unavailability of the experts. Even though this is a descriptive analytical study, a qualitative study or a mixed method study could have assessed the attitude and practices of child abuse better. This study can be generalised to the South Asian region as it involves a large number of responders both from the clinical and community level who belong to the same socio-cultural background.

## Conclusion

The results reveal that the knowledge, attitude and behaviour of the different health care professionals are satisfactory with few deficiencies, mainly in the areas of identifying the perpetrator and the decision they will take in the case of neglect. All the groups felt their knowledge was satisfactory and wanted to further their knowledge through various continuing medical programmes. The experience and professionals involved in child care and judicial work had a statistically significant good knowledge, attitude and behaviour regarding child abuse.

There were barriers in reporting despite a legal requirement; hence support of the child protection services and the effectiveness of these services need to be evaluated. The gap between detecting and reporting can be overcome by improving the knowledge base.

Understanding and clinical competencies in detecting child abuse are crucial knowledge and skills that are required to evaluate the effectiveness of curricula and the programmes involved in CME, in preparing future healthcare professionals to identify, manage and prevent child abuse. A regular check on the outcome of the education has to be assessed and improvements must be made according to latest evidences. Professional education programmes must sensitise all health care professionals of the occurrences and instruct them on how and when to report a suspected case of child abuse and neglect.

## Abbreviations

CME: Continuing medical education
CSA: Child sexual abuse
IMO: Intern medical officer
RHO: Resident house officer
SHO: Senior house officer
WHO: World Health Organisation
